# Analysis of Experiences in Preventing COVID-19 in Hemodialysis Centers of the North of Poland before the Era of Vaccination

**DOI:** 10.3390/ijerph19020684

**Published:** 2022-01-07

**Authors:** Bogdan Biedunkiewicz, Leszek Tylicki, Ewelina Puchalska-Reglińska, Alicja Dębska-Ślizień

**Affiliations:** 1 Department of Nephrology, Transplantology and Internal Medicine, Medical University of Gdańsk, 80-952 Gdańsk, Poland; bogdan.biedunkieiwcz@gumed.edu.pl (B.B.); adeb@gumed.edu.pl (A.D.-Ś.); 2 Dialysis Unit, 7th Naval Hospital in Gdańsk, 80-305 Gdańsk, Poland; e.puchalska@7szmw.pl

**Keywords:** hemodialysis, COVID-19, prevention, epidemiology, incidence

## Abstract

Background: The appearance of very contagious SARS-CoV-2 variants and waning vaccine immunity may indicate the need to return to using universal methods of preventing the spread of COVID-19. Methods: We performed a multicenter retrospective cohort survey study to describe the methods used in dialysis units to prevent and control the spread of SARS-CoV-2 and also the association between these methods and the incidence of COVID-19 among hemodialyzed (HD) patients before the era of vaccination. The study population included all maintenance HD patients (*n* = 1569) in 14 dialysis units in the Pomeranian Voivodeship. Results: The group of 352 patients (199 men, 153 female) were confirmed for COVID-19. The absolute cumulative incidence in the studied period was 22.4%. It varied widely by dialysis units, ranging from 9.4% to 36.9%. Universal preventive methods were applied by all units. Different additional methods were implemented in some stations with varying frequency (36–86%). In order to quantify the scale of the applied additional preventive methods, we calculated a summary prevention index (PI), i.e., one point for one additional method. Lower incidence was found in centers applying dialysis in isolation of patients hospitalized due to diseases requiring hospitalization (17.42% ± 6.89 vs. 26.54 ± 6.34; *p* = 0.028) and higher incidence in medium-size dialysis centers (ANOVA F: *p* = 0.017). Significant inverse correlation between PI and incidence was demonstrated as well (r = −0.759; *p* = 0.002). Conclusions: The higher the number of implemented preventive measures, the lower the risk of COVID-19 infection in HD patients. Among applied procedures the isolation of hospitalized patients is of significant importance. The measures proved to be effective in prevention before the vaccination era should be continued, as the threat of SARS-CoV-2 still exists.

## 1. Introduction

The epidemic of severe acute respiratory syndrome coronavirus 2 (SARS-CoV-2) infection was first reported in December of 2019 in Wuhan (China) [1]. Meanwhile, the virus quickly spread to other regions of mainland China as well as to other places in the world thanks to transport links to the original epicenter [2]. The disease caused by SARS-CoV-2 the World Health Organization (WHO) called “coronavirus disease 19” (COVID-19) and declared it a pandemic on 11 March 2020. The first cases in Poland were reported on 4 March 2020 and on 14 March 2020 in the North of Poland. The first case SARS-CoV-2 in a hemodialysis (HD) patient in the North of Poland (Pomeranian Voivodship) was diagnosed on 7 April 2020 [3]. HD patients have lowered immunity due to uremia, comorbidities, and dialysis procedure-related bio-incompatibility [4]. Furthermore, frequent personal contact in crowded areas for their in-center facility treatment may hamper effective protective measures (such as social distancing, reducing personal contact, staying home) against COVID-19 infection. The dialysis centers became consequently a convenient place for the spread of this disease [5]. It is therefore no surprise that the COVID-19 epidemic has affected a large proportion of HD patients and the incidence was significantly higher than that of the general population [6,7]. All these factors together put HD patients at a high risk of SARS-CoV-2 infection and a severe course of the disease with fatality rates varying from 16% to 42%. Of the hospitalized COVID-19 patients of four dialysis centers in Brescia, Italy, 79% developed acute respiratory distress syndrome (ARDS) and 42% died [8]. In our previous study we showed the extremely high mortality of COVID-19 in HD patients from the North of Poland, with a fatality rate up to 43.81% in subjects over 74 years of age [7].

In line with the guidelines, in the first phase of the pandemic, dialysis center personnel attempted to identify; isolate; and, where possible, investigate patients suspected of being infected with SARS-CoV-2. The universal recommendations for dialysis centers, aimed to limit the spread of the disease among patients and staff, were developed by different health authorities and scientific societies [9]. Additionally, various additional preventive measures were introduced and applied by individual dialysis centers. We set out to identify methods of preventing infections with SARS-CoV-2 that may affect the incidence of COVID-19 in maintenance HD patients. In the period the survey was carried out, dialyzed patients were not yet vaccinated in Poland. The process of vaccination was initiated in late December 2020 in medical staff and in late January 2021 for dialysis patients.

## 2. Materials and Methods

### 2.1. Study Protocol

Study design—a multicenter retrospective study. The primary outcome was to identify methods used in dialysis units to prevent and control the spread of SARS-CoV-2 among HD patients. A secondary outcome was to find the association between these methods and the incidence of SARS-CoV-2 infection. Ethics approval for the study in all dialysis units was obtained at the Medical University of Gdańsk (NKBBN/2014/2021). The study is part of the “COVID-19 in Nephrology” (COViNEPH) project focusing on the nephrological aspects of COVID-19, in particular epidemiology, prevention, disease course, and treatment.

### 2.2. Study Subjects and Methods

The study population included all prevalent adult patients from 14 dialysis units in the Pomeranian Voivodeship reported to the Polish Registry on 31 December 2019 and all new patients starting long-term hemodialysis between 1 January 2020 and 31 January 2021 (*n* = 1569) [10,11]. Each dialysis center was asked to report all new cases of COVID-19 due to the disease since the beginning of the pandemic until 31 January 2021, as well as to describe the methods applied in the unit to prevent and limit SARS-CoV-2 infection spread. Cases were considered confirmed if they had laboratory isolation of the SARS-CoV-2 by an RT-PCR test from nasopharyngeal/oropharyngeal swabs. To find factors affecting COVID-19 incidence in dialysis centers, they were categorized as public and non-public (3 and 11, respectively); small (<75 pts)—4 centers, medium (75–150 pts)—5 centers, and large (>150 pts)—5 centers; and those that treat with hemodialysis both uninfected and infected with SARS-CoV-2 patients—4 centers and those treating only non-infected patients—10 centers. In the province, there was one dialysis center dedicated only for patients infected with SARS-CoV-2.

The following preventive methods were considered universal: wearing protective masks by patients and medical staff all the time while in the station, an epidemiological interview related to COVID-19 symptoms before entering the dialysis station, and body temperature measurement at home and before entering the dialysis station. In addition, in order to quantify the scale of the applied additional preventive methods, the summary prevention index (PI) was calculated (one point for one additional method, range: 0–6), counting the following activities: (a) hemodialysis treatment in isolation for patients after contact with individuals suspected of COVID-19 or infected with SARS-CoV-2; (b) prohibition of eating and drinking by patients throughout their stay at the dialysis station; (c) prohibition of eating meals together by the medical staff in the unit (maximum 1 person eating alone in the room); (d) hemodialysis treatment in isolation for patient after hospitalization; (e) hemodialysis treatment in isolation for currently hospitalized patients for any reasons; (f) periodic screening of all staff for the presence of SARS-CoV-2 infection. Isolated hemodialysis treatment for patients after contact with SARS-CoV-2-infected or with COVID-19 suspected persons as well as after hospitalization was usually discontinued after 10 days of isolation and negative RT-PCR test (from nasopharyngeal/oropharyngeal swabs). The centers avoided mixing suspected and confirmed cases while waiting for a swab response. Patients with a positive RT-PCR test were transferred to a dedicated dialysis station for COVID-19 patients. In four centers, they were treated in isolation (separate shift and room). Screening of all staff for the presence of SARS-CoV-2 infection at selected dialysis centers took place when infection was confirmed in any team member. Other rare preventive methods used by single dialysis centers were recorded as well.

### 2.3. Statistical Analyses

Descriptive statistics were used to characterize dialysis units and to describe the preventive methods applied by them. Continuous variables were presented as mean ± SD. Student’s *t*-test or Mann–Whitney *U* test was used to compare continuous variables. The differences in the incidence between the three types of dialysis stations depending on their size were analyzed using analysis of variance (ANOVA F). Categorical variables were presented as relative and absolute frequency; a chi-squared test or Fisher’s exact test analysis was used as appropriate to compare categorical variables. A two-sided *p*-value of <0.05 was considered significant.

## 3. Results

In total, 100% of dialysis units provided their data. The total number of exposed HD patients in the studied period was 1569. Of these, 352 (199 m, 153 f) patients were confirmed for COVID-19. The incidence rate was calculated as follows: (the number of new cases from 14 March 2020 until 31 January 2021/number of exposed people) × 100. The absolute cumulative incidence in the studied period was 22.4%. It varied widely by dialysis units, ranging from 9.4% to 36.9% (Table 1). The fatality rate ranged between dialysis units from 11.1% to 58.8% (Table 1). There were no correlations between fatality rate vs. COVID-19 incidence and PI.

Universal preventive methods were applied by all units. Different additional methods were implemented in some stations with varying frequency (36–86%) (Table 2). Of these, the most often used were as follows: hemodialysis in isolation for patient after contact with persons with COVID-19 or with those suspected of having COVID-19, and restriction of shared food consumption by staff (Table 2). Individual stations used their own developed prevalence methods, typical for a given center. Separate entrances and exits for patients in order to limit the contact of patients with various shifts were used in two stations. Division of health staff into two nursing-medical teams dealing with different groups of patients was temporarily introduced in one station. Another one conducted telephone epidemiological interviews with patients that preceded each of their arrivals to the dialysis center. In some units, patients were informed about the potential epidemiological advantages of using their own transport to and from the HD unit.

**Figure 1:**
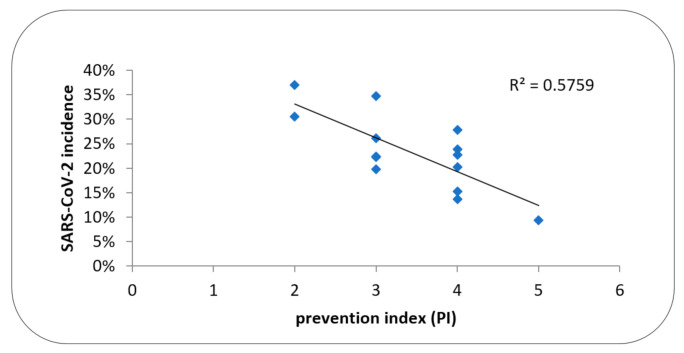
The relationship between PI and rate of SARS-CoV-2 incidence (%).

Significant inverse correlation between the PI index and COVID-19 incidence in HD stations was demonstrated (r = −0.759; *p* = 0.002) (Figure 1). In strata analyses, significantly lower incidence was found in HD centers applying isolation of hospitalized patients (isolation in a separate room or separate shift) (17.42 ± 6.89 vs. 26.54 ± 6.34; *p* = 0.028) (Table 3). The highest incidence of 30.4% ± 96.4 was found in the medium-size dialysis centers as compared to small and large HD units (19.83% ± 6.63 and 18.83% ± 5.42) (ANOVA F: *p* = 0.017) (Table 3). The medium size centers presented with the lowest index PI (2.8 ± 0.84 vs.4.0 ± 0.0 vs. 3.6 ± 0.89, respectively) (ANOVA: *p* = 0.083). There were no differences in the incidence (23.98 ± 7.64 vs. 21.53 ± 8.88%) and in the PI index (3.4 ± 0.69 vs. 3.5 ± 1.29) between HD centers that dialyzed and did not dialyze patients infected with SARS-CoV-2.

## 4. Discussion

In our previous study, we showed the very fatality rate of SARS-CoV-2 infection among HD patients from the Pomeranian Voivodeship [7]. This study confirms previous findings that chronic HD patients are at a high risk of developing COVID-19 [12,13,14,15]. Moreover, we confirm that the implementation of preventive structural and organizational changes applied to all patients and healthcare personnel may decrease the risk of local transmission of SARS-CoV-2 infection [16,17]. We demonstrated that COVID-19 incidence varied widely by dialysis units from 9.4% to 36.4%; thus, one may hypothesize these variations may be due to the too-liberal policy adopted by some units with regard to safety measures. Generally, the HD units participating in the survey followed the recommendations for the universal prevention of SARS-CoV-2 infections proposed by the National Consultant for Nephrology. Importantly, we confirmed that the use of additional preventive measures reduces the spread of the virus even in units dialyzing both noninfected and infected patients. On the other hand, in medium-sized stations that used the least additional preventive methods, we observed the most infections. Among the additional methods introduced by dialysis stations, isolation of hospitalized patients for any reason during their stay in a dialysis station had a significant impact on reducing the incidence. The idea behind such action resulted from observations of local infection foci in wards, from which the infection was transferred to other wards. Generally, the more methods, the better protection of patients.

**Figure 2:**
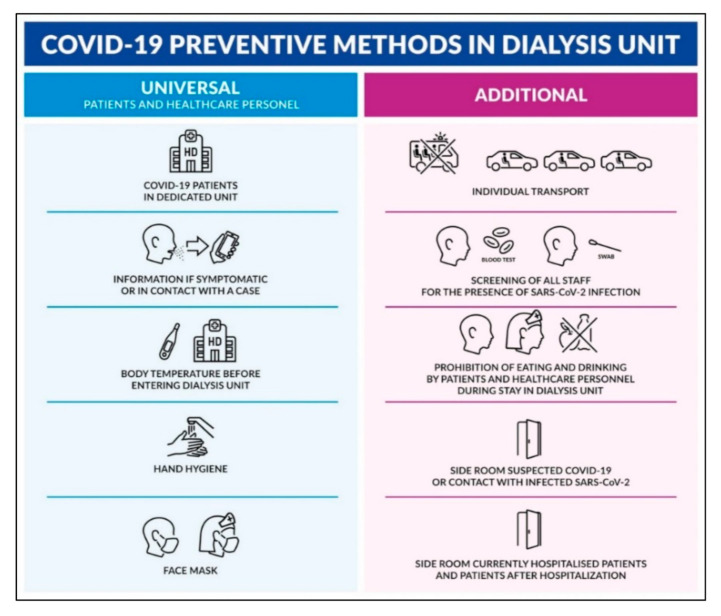
Recommendations for COVID-19 preventive methods in a dialysis unit.

The appearance of a very contagious SARS-CoV-2 delta variant in recent months, despite successful vaccination in most patients, may indicate the need to return to using universal methods of preventing the spread of COVID-19. This is confirmed by breakthrough infection in fully vaccinated individuals [18,19]. The validity of the use of universal preventive methods is confirmed, for example, by studies on the use of masks to prevent COVID-19 in the general population, including fully vaccinated people [20,21]. Therefore, the recommendation of the use of masks to prevent COVID-19 in dialyzed patients should stay until the end of the epidemic, independently of its magnitude and of vaccination. There is also no doubt that other common preventive methods such as disinfection of the dialysis monitor, chair/bed, table, and blood pressure monitor after each HD treatment; division of health staff into two nursing-medical teams; limiting the contacts of patients in the changing rooms and waiting rooms; efficient ventilation of dialysis rooms; and the use of disposable uniforms by patients during their stay in the station should take place as far as possible at the HD unit. It cannot be ruled out that noncompliance of some patients, or single cases of careless behavior, may account for the observed differences in the incidence of SARS-CoV-2 infection between HD units. SARS-CoV-2 is highly infectious, and a single error can result in the uncontrolled spread of COVID-19 among patients and dialysis center staff. One of the factors not analyzed in our study that could have significantly influenced the differences in incidence is the joint transport of dialysis patients. Patients going to dialysis centers often travel long distances together and spend a long time in the company of themselves, which increases the risk of infection and transmitting the virus. Therefore, the recommendation to avoid public transport and to use your own transport is still valid [9,15,22]. Taking into account the results of the study and our own clinical experience form the medium-sized hospital HD station, we recommend the preventive procedures as demonstrated in the Figure 2.

An important issue in the prevention of COVID-19 in dialysis stations, and not addressed in this analysis, is the fastest possible diagnosis of infections. The available SARS-CoV-2 diagnostic tests do not always confirm the diagnosis of an infection; therefore, it becomes essential to have accurate tools for the diagnosis of COVID-19. Each dialysis center should implement local strategies for the early recognition of COVID-19 patients. A holistic diagnostic approach (medical history, exposure risk, clinical examination, laboratory examination, CT, and ultrasound of the lungs) can increase the diagnostic accuracy of COVID-19 [23,24], especially in mild-to-moderate disease [25].

Our work has a number of limitations, including the observational design that limits the conclusions that can be drawn about causality. Data were collected retrospectively, on the basis of questions from questionnaires, which despite the validation controls performed, could be interpreted differently, depending on the center. The calculated summary PI counting several preventive activities. However, the selection of these preventive activities was arbitrary. The vaccination as a preventive activity was not available at the moment of the study. The study was conducted before the SARS-CoV-2 vaccination era, and none of the patients was vaccinated at the time of the study. We did not analyze the characteristics of the patients treated in the units, which could of course have an impact on the results of the analyzes.

## 5. Conclusions

In conclusion, it should be emphasized that the more methods of preventing SARS-CoV-2 infection we introduce in dialysis centers, the more we reduce the risk of an increased incidence of COVID-19 among our patients. Additional procedures, individually introduced by centers, often taking into account their specificity, such as isolation of hospitalized patients in a separate room or separate shift, are also important. Consequent applying of the mentioned protective measures may also allow the dialysis in one unit of both infected and noninfected patients without an increased risk of infection.

## Figures and Tables

**Table 1:** SARS-CoV-2 infection incidence in the hemodialysis centers of the Pomeranian Voivodeship.

HD Center	Size and Status of HD Center	HD Patients 31.12.2019 (*n*)	HD pts in the Study (*n*)	Incidence %	Fatality Rate %	PI
HDC001	2P	74	92	36.4	32.3	2
HDC002	2NP	78	89	34.8	12.9	3
HDC003 *	2NP	68	85	30.6	34.6	2
HDC004	1NP	56	72	27.8	35.0	4
HDC005	2P	85	103	26.2	40.7	3
HDC006 *	2NP	83	109	23.8	30.8	4
HDC007	1NP	53	66	22.7	46.7	4
HDC008 *	3NP	144	206	22.3	19.6	2
HDC009	3NP	109	139	22.3	38.7	3
HDC010	3NP	146	177	20.3	25.0	4
HDC011	3P	78	131	19.8	19.2	3
HDC012	1NP	41	59	15.2	11.1	4
HDC013	1NP	45	59	13.6	50.0	4
HDC014 *	3NP	132	182	9.4	58.8	5

HD center: NP—non-public, P—public. Size of the HD center: 1—<75 pts, 2—75–150 pts, 3—>150 pts. PI—Prevention Index (range: 0–6). *—centers that treat with hemodialysis both uninfected and infected with SARS-CoV-2 patients.

**Table 2:** Prevalence of universal and additional prevention methods in HD centers.

Preventive Method	U1 ^a^	U2 ^a^	U3 ^a^	A1	A2	A3	A4	A5	A6
Number of HD centers	14	14	14	10	9	12	5	5	6
%	100%	100%	100%	71%	64%	86%	36%	36%	43%

^a^—universal prevention methods (shaded gray). U—universal prevention methods: U1—protective mask for medical staff; U2—protective mask for the patients; U3—medical history, temperature measurement, social distance. A—additional prevention methods: A1—no meal and drink for patients in HD center; A2—no common meal and drink for medical staff in HD center; A3—dialysis in isolation for patients after contact with COVID-19 subject or with suspected for SARS-CoV-2 infection; A4—hospitalized patients dialyzed in isolation; A5—patients after hospitalization dialyzed in isolation; A6—medical staff PCR-RT swab screening after exposure.

**Table 3:** The incidence of SARS-CoV-2 infection in strata analyses.

Parameter	*n*	Incidence (%)	*p*
Public HD center	4	26.33 ± 7.55	ns
Non-public HD center	10	22.06 ± 7.56	
HD center 1 (small size) ^a^	4	19.83 ± 6.63	0.017
HD center 2 (medium size)	5	30.49 ± 6.4 ^b^	
HD center 3 (large size)	5	18.83 ± 5.42	
COVID-19-positive patients in HD center	4	19.33 ± 5.95	ns
Only COVID-19-negative patients in HD center	10	24.86 ± 8.06	
Suspected patient isolation	12	21.33 ± 1.41	ns
Suspected patients without isolation	2	29.35 ± 7.71	
Isolation after hospitalization	5	22.12 ± 9.13	ns
No isolation after hospitalization	9	23.92 ± 7.37	
Isolation of hospitalized patients	5	17.41 ± 6.90	0.028
Hospitalized patients without isolation	9	26.54 ± 6.34	
Screening of all staff (after exposure)	6	22.25 ± 8.05	ns
Without screening of all staff (after exposure)	8	24.90 ± 7.36	
Staff not eating and drinking together	9	21.90 ± 7.39	ns
Staff eating and drinking together	5	25.77 ± 8.58	
Patients not eating and drinking in HD center	10	23.32 ± 8.31	ns
Patients eating and drinking in HD center	4	23.17 ± 7.24	

^a^—size of the HD center: 1—<75 pts, 2—75–150 pts, 3—>150; ns—non significant. ^b^—medium vs. small and large size HD center.

## Data Availability

Detailed data are available on request from corresponding author.
